# Notable Stabilization of *α*-Chymotrypsin by the Protic Ionic Additive, [ch][dhp]: Calorimetric Evidence for a Fine Enthalpy/Entropy Balance

**DOI:** 10.1155/2014/834189

**Published:** 2014-09-07

**Authors:** Sophio Uchaneishvili, Maya Makharadze, Mikhael Shushanyan, Rudi van Eldik, Dimitri E. Khoshtariya

**Affiliations:** ^1^Department of Biophysics, I. Beritashvili Center of Experimental Biomedicine, Gotua 14, 0160 Tbilisi, Georgia; ^2^Institute for Biophysics and Bionanosciences at the Department of Physics, I. Javakhishvili Tbilisi State University, I. Chavchavadze Avenue 3, 0128 Tbilisi, Georgia; ^3^Department of Chemistry and Pharmacy, Friedrich-Alexander University of Erlangen-Nürnberg, Egerlandstraße 1, 91058 Erlangen, Germany

## Abstract

An impact of 0.5 to 3 M choline dihydrogen phosphate, [ch][dhp], the biotechnologically relevant ionic substance, on the thermal stability of a model globular protein, α-chymotrypsin (α-CT), has been studied exploiting the highly sensitive differential scanning calorimetry (DSC) technique. The notable overall stabilizing effect of 11 ± 2 K regarding the thermal transition (melting) temperature, *T*_*m*_, has been detected. For this kind of series, for the first time, the calorimetric melting enthalpy (Δ*H*_cal_) and transition entropy (Δ*S*_*m*_) parameters have been determined simultaneously throughout. The first analysis indicated a two-phase impact implying (a) the initial, dramatic drop in both Δ*H*_cal_ and Δ*S*_*m*_, obviously connected to specific, direct interaction between the [ch][dhp] components and α-CT's charged groups (within 0 to 1 mol/L [ch][dhp]), leading to the essential rearrangement of the interfacial hydrogen-bonded (HB) network; and (b) the follow-up (within 1 to 3.0 mol/L [ch][dhp]), modest changes in Δ*H*_cal_ and lack of changes in Δ*S*_*m*_, seemingly connected with a subsequent steady strengthening of already reformed HB network, respectively. These changes, presumably, are primarily facilitated by Coulombic interactions between the [dhp] anions and solvent-exposed positively charged amino groups of α-CT.

## 1. Introduction

Currently, there is a great interest in the multipurpose application of recently revived substances, organic salts possessing relatively low melting points (also known as “room-temperature ionic liquids,” RTILs, or “plastic ionic crystals”). These substances, when mixed with water, are able to form a huge amount of unique liquid or semisolid compositions apt for the novel nanotechnological and bionanotechnological developments [1–4]. Because of virtually unlimited possibilities for the variation of an intrinsic molecular balance between the charged and uncharged (hydrophobic) groups on both positive and negative ionic components [1], these substances may offer potentially ideal milieu for any kind of reactant species including biomolecules (e.g., globular proteins), assembled in any kind of (bio)nanodevice. For the latter case, the major point, actually, implies design of optimal environmental conditions for the improved stability, activity, and refolding ability of “working” elements such as proteins, for example [4–22]. Furthermore, introduction of protic RTILs, thanks to their essential protonic conductivity, offers superior conditions for the proper functioning of bioinspired nanovoltaic cells/devices [1, 3, 22].

As to the stability issue for globular proteins under the impact of different additives, including RTILs, it can be adequately approached by the method of differential scanning calorimetry (DSC) [4, 5, 8–11, 13–17, 23–26]. Specifically, the DSC technique detects the temperature-induced unfolding (melting) process (associated with a global transition along the protein's collective conformational coordinate) as a variation of the protein's partial heat capacity versus temperature and, in uncomplicated cases, in a model-free way (directly) determines the unique “transition temperature” (temperature of thermal melting), *T*_*m*_, the unfolding (melting) calorimetric enthalpy, Δ*H*_cal_ (i.e., the heat absorbed through the unfolding transformation around *T*_*m*_), and calorimetric entropy, Δ*S*_cal_ [27–29].

Numerous papers on DSC studies for the stabilizing/destabilizing impact of various organic additives [4, 5, 8–11, 13–17, 23–26, 30], including RTILs [4, 5, 8–11, 13–17], on the proteins' global stability have been published. However, because of a complex, dualistic character of the RTIL impact (due to the involvement of oppositely charged surface-active ions), notwithstanding of several serious attempts [16, 17, 21], no conclusive rationalization of the existing data was drawn so far. Obviously, more systematic and careful work with the application of the DSC combined with other methodologies is required. Indeed, in many cases of serial DSC studies implying the RTIL impact, typically, only the parameter *T*_*m*_, as a measure of stability, has been considered; the easily calculable parameter Δ*H*_cal_ has been addressed in very few cases (see, e.g., [5, 8, 9]; for other similar studies involving uncharged additives, see, e.g., [4, 23, 26, 30]). It is instructive that another easily calculable parameter, Δ*S*_cal_ (vide infra), has never been implicated for this kind of series. An entropic contribution is equally important for the understanding of the protein stability phenomenon, as its enthalpic counterpart [29, 31–33]. In fact, stability of the native state results from the fine balance between a multitude of the enthalpic and entropic contributing factors (vide infra) [4, 27, 30, 31, 33]. Among other model proteins, in which thermal stability has been extensively explored by the DSC technique, α-chymotrypsin (α-CT, EC 3.4.21.1; *M*_*r*_ = 26,000), a hydrolytic enzyme of biotechnological importance, should be mentioned [4, 5, 23, 24, 26, 30]. However, detailed studies regarding the impact of RTILs on α-CT, using highly sensitive DSC technique (implying determination and analysis of all the key parameters, *T*_*m*_, Δ*H*_cal_, and Δ*S*_cal_), are still lacking. In this communication, we report preliminary data and principal thermodynamic analysis on the stabilizing impact of biotechnologically important concentrations of a protic ionic salt, choline dihydrogen phosphate, [ch][dhp], on the thermal stability of model protein, α-CT. Determination of both enthalpic and entropic components contributing to the protein melting process allowed for a conjecturing of fine interfacial effects behind the notable stabilization impact.

## 2. Experiment

Highly purified and lyophilized α-CT was purchased from Fluka and [ch][dhp] was from Iolitech, GmbH, and used without further purification. Other chemicals were of highest purity available. The α-CT samples for the DSC experiments were prepared at concentrations of 1.5 mg/mL, by dissolving in phosphate 10^−2 ^M buffer solutions (pH 4.2).

Microcalorimetric measurements for temperature-induced melting (denaturation) of α-CT in buffered [ch][dhp] aqueous solutions were performed with a DASM-4A adiabatic scanning calorimeter (Biopribor, Russia) integrated with a PCI-DAS1001 (Measurement Computing Corporation) interface unit, providing direct PC access [30]. The heating rate was 2 K/min. The methodological aspects of the DSC technique and data processing have been described earlier [23, 27, 28, 30].

## 3. Results and Discussion

**Figure 1:**
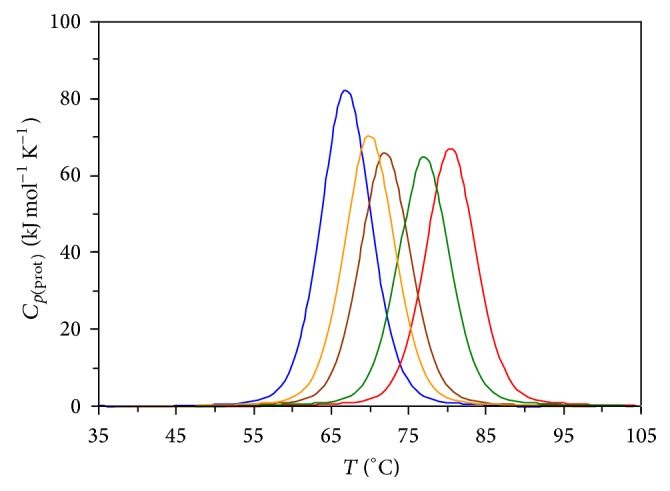
The DSC curves for the α-CT thermal unfolding in the absence and presence of 0.5 to 3 M [ch][dhp] concentrations; pH 4.2 (phosphate buffer).

**Figure 2:**
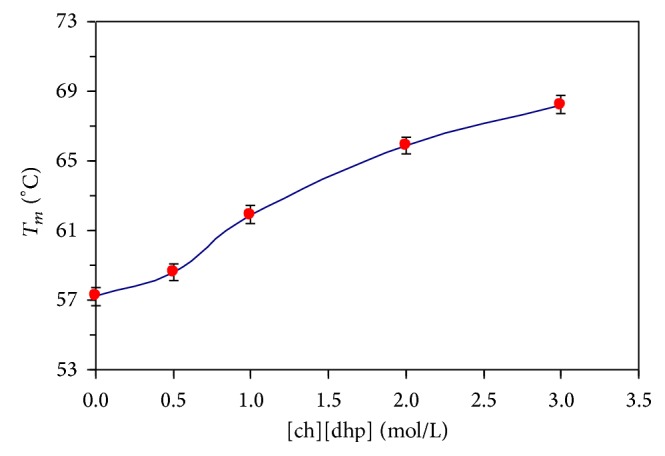
Dependencies of the transition temperatures for the temperature-induced melting of α-CT on the [ch][dhp] concentration (0 to 3 M; pH 4.2).

**Figure 3:**
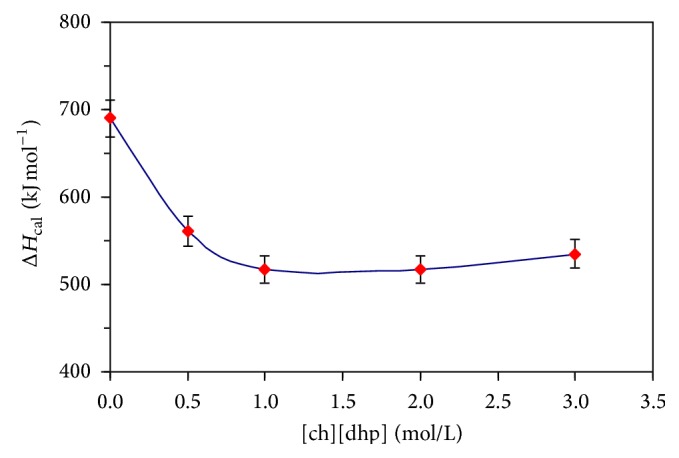
Dependence of the calorimetric enthalpy for temperature-induced melting of α-CT on the [ch][dhp] concentration (0 to 3 M; pH 4.2).

**Figure 4:**
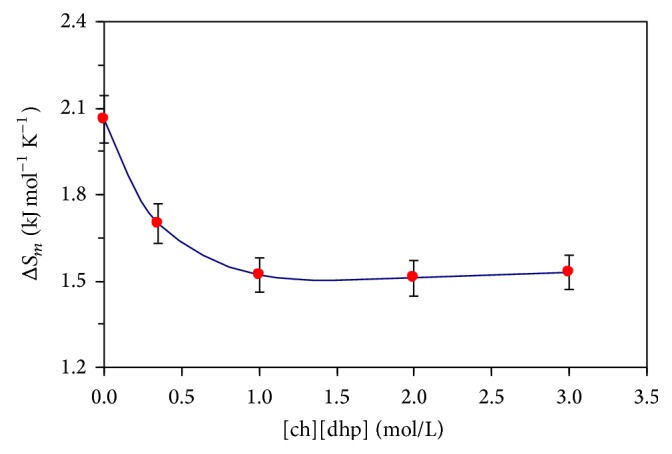
Dependence of the transition entropy for temperature-induced melting of α-CT on the [ch][dhp] concentration (0 to 3 M; pH 4.2).

Figure 1 depicts the zero-baseline-corrected calorimetric curves (partial heat capacity of protein versus temperature) for the temperature-induced melting of α-CT in the absence and presence of the [ch][dhp] additives, 0, 0.5, 1.0, 2.0, and 3.0 mol/l. Accordingly, Figures 2, 3, and 4 and Table 1 depict results of thermodynamic analysis of these melting curves. The values for a melting enthalpy, Δ*H*_cal_, were determined as areas under the calorimetric curves of Figure 1 [23, 27–30]:

(1) $$\begin{array}{c} \Delta H_{\text{cal}}=\overset{T_{2}}{\underset{T_{1}}{\int }}C_{p(\text{prot})}\;dT, \end{array}$$

where *T* is the absolute temperature and *T*_1_ and *T*_2_ are the temperatures that correspond to the start and completion of heat absorption due to the protein's thermal melting (*T*_1_ < *T*_*m*_ < *T*_2_). The values of the counterpart parameter, melting entropy, Δ*S*_cal_, can be determined as follows [29]:

(2) $$\begin{array}{c} \Delta S_{\text{cal}}=\overset{T_{2}}{\underset{T_{1}}{\int }}\begin{array}{c} \frac{C_{p(\text{prot})}}{T}\;dT. \end{array} \end{array}$$

Under the assumption that a two-state model for the protein melting process is operative, that is, (a) at *T*_*m*_ concentrations of the native and melted (unfolded) protein are equal and (b) virtually the entire entropy change takes place at this temperature (therefore, Δ*G*_*m*_ = Δ*H*_cal_ − *T*_*m*_Δ*S*_*m*_ = 0), (2) is simplified as follows:

(3) $$\begin{array}{c} \Delta S_{m}=\frac{\Delta H_{\text{cal}}}{T_{m}}. \end{array}$$

However, one may question whether (1) to (3) and related analysis (vide infra) are valid for the case of α-CT, known as a protein in which thermal unfolding process, in general, deviates from a simple two-state pattern (see, e.g., [24, 30]). Rather detailed analysis of this issue is given in our previous paper [30] (see also [17, 34]). In brief, in the course of a thermal melting of most globular proteins of modest dimensions, including α-CT, the native and “normally” unfolded states can be considered as yet dominantly populated states against the relatively minor, thermally induced molten globule (predenatured) [25, 34], “intermediate” (metastable transitional) [16, 34], and misfolded/self-aggregated (postdenatured) states [16, 29, 35]. Importantly, the DSC curves retained symmetrical, nearly Gaussian shape (half widths were within 6.7 ± 0.2 K throughout) upon the addition of 0.5 to 3 mol/l [ch][dhp] (the fact that favors the abovementioned outline, yet, as a crude approximation [30]), whilst their maximum position (*T*_*m*_) was gradually shifted to higher temperatures (for ca. 11 K, in overall), pointing to the notable stabilizing impact of [ch][dhp] (Table 1). The values of Δ*S*_cal_ (attained for selected cases) and Δ*S*_*m*_, as determined through (2) and (3), coincided within the limits of experimental error, that is, an additional support for the practical applicability of (3). The slope for the first change of *T*_*m*_ versus the [ch][dhp]) content (from 0 to 1 mol/l [ch][dhp]) was a little bit larger than one for the rest of the concentration range (Figure 2). This fact could be ascribable to the experimental scatter of experimental points if there is no remarkable and drastic mutually compensating change of both Δ*H*_cal_ and Δ*S*_*m*_ resulting only in a modest shift of *T*_*m*_ (Figures 2 to 4, Table 1). Next, in a concentration range from 1 to 3 mol/l [ch][dhp], parameter Δ*H*_cal_ increased gradually very little, whereas parameter Δ*S*_*m*_ remained unchanged within the limits of experimental error. This occurrence allowed parameter *T*_*m*_ to increase further steadily (Figure 2) (vide infra).

Interestingly, Constatinescu et al. [11] recently reported on ca. 16 K increase in the melting temperature of another model protein, ribonuclease A, upon the adding of 3 mol/L [ch][dhp] (overall, ≈20 K increase upon the adding of 4 mol/L [ch][dhp]). The shift values for *T*_*m*_ and a trend for the dependence of *T*_*m*_ on the [ch][dhp] content are very similar to ours, having the distinct slope break at 0.5 mol/L (see Figure 2 of [11]). However, as can be judged based on the incomplete Figure 1 of [14], the increase in *T*_*m*_ was accompanied by the substantial increase in Δ*H*_cal_. Unfortunately, the whole trend for Δ*H*_cal_ (and, even more so, for Δ*S*_*m*_) is not reported in [11]. In another recent work, Rodrigues et al. [15] reported on ca. 11 K increase in a melting temperature of the third model protein, lysozyme, upon the adding of 2.5 mol/L [ch][dhp]. The overall effect is absolutely similar to ours (implying the interpolated value of *T*_*m*_ ≈ 74°C at 2.5 mol/L [ch][dhp] for our system); however, the overall dependence of *T*_*m*_ on the [ch][dhp] content for lysozyme, in contrast to our case (Figure 1), exhibited well-pronounced minimum at 0.5 mol/L [ch][dhp]. Unfortunately, again, the authors of [15] did not report the values for Δ*H*_cal_ and Δ*S*_cal_ (Δ*S*_*m*_) at any concentration of the additive. The lacking information would allow one to see the variable balance between Δ*H*_cal_ and Δ*S*_*m*_ behind the resultant behavior of *T*_*m*_ in these studies, hence, to compare the complete data for all three model proteins and gain better insight into atomistic mechanisms of stabilization.

Nonetheless, the first analysis of our results together with comparable published data [11, 15] permits presuming that the mechanisms behind the initial drop in values of both Δ*H*_cal_ and Δ*S*_*m*_ and further slight increase with and virtual independence of *T*_*m*_, respectively, are obviously different. When going from zero to 0.5 ÷ 1 mol/L additive concentration, seemingly, strong association between the [ch][dhp] components and proteins' charged groups takes place that should be rather specific to the protein entity and its ionization state (pH); note different patterns of *T*_*m*_ for α-CT and ribonuclease A on the one hand and lysozyme on the other hand (note also different Δ*H*_cal_ patterns for α-CT and ribonuclease A, mentioned above). Around this point of the additive concentration, the specific interactions, probably, arrive at the saturation level, after which more gradual/monotonic changes of *T*_*m*_ (in all three cases) and very small/no variation of Δ*H*_cal_ and Δ*S*_*m*_ (as detected in our case) can be observed. The small changes in Δ*H*_cal_, seemingly, are connected with the formation of additional contacts and some redistribution of existing ones between the protein and the [ch][dhp] components. Development of the latter process seems to favor proteins' gradual stabilization via the Δ*H*_cal_ rather than the Δ*S*_*m*_ parameter. According to the analysis of [16] (the most systematic one, so far), the choline cation is deemed to act as a slightly destabilizing component whereas dihydrogen phosphate anion is known as an efficient stabilizer. In addition, this pattern turned to be dependent on the additive concentration and has to be also essentially pH dependent [16]. Hence, around pH 4.2, where the exposed amino groups are 100% positively charged, the [dhp] anions, able to effectively interact with these groups through the Coulombic attraction, may eventually replace the existing hydrogen-bond networks originally formed by solvating water and contribute to the protein stabilization. The enthalpic rather entropic nature of the stabilization impact of [ch][dhp] above 1 mol/L concentration definitely points to a prevailing role of the hydrogen-bond network strengthening rather than that of additional strengthening of electrostatic interactions (between any kind of the “point charges” involved). At this pH, the surface exposed carboxylate groups of α-CT are for only ca. 50% negatively charged; therefore, respective Coulombic and associated hydrogen-bonding interactions may play relatively minor role. Further systematic work is in progress aiming at more comprehensive insights at the atomistic level.

## 4. Conclusions

(a) We applied the very sensitive DSC technique to study an impact of 0.5 to 3 mol/L additives of the biotechnologically relevant ionic substance [ch][dhp] on the thermal stability of a model globular protein α-CT and found the notable stabilizing effect of 11 ± 2 K regarding the thermal unfolding (melting) temperature, *T*_*m*_, that varied monotonically with the additive concentration.

(b) The calorimetric melting enthalpy (Δ*H*_cal_) and transition entropy (Δ*S*_*m*_) parameters were determined simultaneously throughout the series, and the trend analysis indicated a two-phase pattern including the low-concentration (0 to 1 mol/L [ch][dhp]) and high-concentration (1 to 3 mol/L [ch][dhp])) regions, connected to the initial dramatic and the follow-up minor or no changes of Δ*H*_cal_ and Δ*S*_*m*_, respectively.

(c) The atomistic mechanism behind the observed overall stabilizing effect, presumably, is guided by the ion pair formation between the protein's charged groups and the [ch][dhp] components, with a prevailing role of restructured hydrogen-bonded networks facilitated mostly by Coulombic interaction between the [dhp] anions and solvent-exposed positively charged amino groups of α-CT.

(d) A need for the determination of parameters Δ*H*_cal_ and Δ*S*_cal_ (Δ*S*_*m*_) throughout this kind of series, for the proper understanding of atomistic mechanisms behind the stabilizing/destabilizing impact of various additives on proteins, is clearly demonstrated.

## Figures and Tables

**Table 1:** Thermodynamic parameters for the thermal unfolding of the *α*-CT in the presence of different [ch][dhp] concentrations phosphate buffer, pH 4.2.

[ch][dhp], mol/L	[ch][dhp], % (w/w)	*T*_*m*_, °C (K)	Δ*H*_cal_, kJ mol^−1^	Δ*S*_*m*_, kJ mol^−1^ K^−1^
0	0	62.8 (335.8)	690	2.06
0.5	12.5	65.8 (338.8)	560	1.70
1	25	67.7 (340.7)	517	1.52
2	50	72.1 (345.1)	520	1.51
3	75	76.0 (349.0)	535	1.53

## References

[1] Angell C. A., Byrne N., Belieres J. (2007). Parallel developments in aprotic and protic ionic liquids: physical chemistry and applications. *Accounts of Chemical Research*.

[2] van Rantwijk F., Sheldon R. A. (2007). Biocatalysis in ionic liquids. *Chemical Reviews*.

[3] Rana U. A., Bayley P. M., Vijayaraghavan R., Howlett P., MacFarlane D. R., Forsyth M. (2010). Proton transport in choline dihydrogen phosphate/H_3_PO_4_ mixtures. *Physical Chemistry Chemical Physics*.

[4] Kumar A., Venkatesu P. (2012). Overview of the stability of α-chymotrypsin in different solvent media. *Chemical Reviews*.

[5] De Diego T., Lozano P., Gmouh S., Vaultier M., Iborra J. L. (2004). Fluorescence and CD spectroscopic analysis of the α-chymotrypsin stabilization by the ionic liquid, 1-ethyl-3-methylimidazolium bis[(trifluoromethyl)sulfonyl]amide. *Biotechnology and Bioengineering*.

[6] Fujita K., MacFarlane D. R., Forsyth M. (2005). Protein solubilising and stabilising ionic liquids. *Chemical Communications*.

[7] Fujita K., Ohno H. (2010). Enzymatic activity and thermal stability of metallo proteins in hydrated ionic liquids. *Biopolymers*.

[8] Byrne N., Angell C. A. (2008). Protein unfolding, and the “tuning in” of reversible intermediate states, in protic ionic liquid media. *Journal of Molecular Biology*.

[9] Byrne N., Belieres J. P., Angell C. A. (2009). The “refoldability” of selected proteins in ionic liquids as a stabilization criterion, leading to a conjecture on biogenesis. *Australian Journal of Chemistry*.

[10] Buchfink R., Tischer A., Patil G., Rudolph R., Lange C. (2010). Ionic liquids as refolding additives: variation of the anion. *Journal of Biotechnology*.

[11] Constatinescu D., Herrmann C., Weingärtner H. (2010). Patterns of protein unfolding and protein aggregation in ionic liquids. *Physical Chemistry Chemical Physics*.

[12] Attri P., Venkatesu P., Kumar A., Byrne N. (2011). A protic ionic liquid attenuates the deleterious actions of urea on *α*-chymotrypsin. *Physical Chemistry Chemical Physics*.

[13] Yamamoto E., Yamaguchi S., Nagamune T. (2011). Protein refolding by N-alkylpyridinium and n-alkyl-n-methylpyrrolidinium ionic liquids. *Applied Biochemistry and Biotechnology*.

[14] Dabirmanesh B., Daneshjou S., Sepahi A. A. (2011). Effect of ionic liquids on the structure, stability and activity of two related α-amylases. *International Journal of Biological Macromolecules*.

[15] Rodrigues J. V., Prosinecki V., Marrucho I., Rebelo L. P. N., Gomes C. M. (2011). Protein stability in an ionic liquid milieu: on the use of differential scanning fluorimetry. *Physical Chemistry Chemical Physics*.

[16] Weingärtner H., Cabrele C., Herrmann C. (2012). How ionic liquids can help to stabilize native proteins. *Physical Chemistry Chemical Physics*.

[17] Figueiredo A. M., Sardinha J., Mooreb G. R., Cabrita E. J. (2013). Protein destabilisation in ionic liquids: the role of preferential interactions in denaturation. *Physical Chemistry and Chemical Physics*.

[18] Nordwald E. M., Kaar J. L. (2013). Mediating electrostatic binding of 1-butyl-3-methylimidazolium chloride to enzyme surfaces improves conformational stability. *The Journal of Physical Chemistry B*.

[19] Nordwald E. M., Kaar J. L. (2013). Stabilization of enzymes in ionic liquids via modification of enzyme charge. *Biotechnology and Bioengineering*.

[20] Latif M. A. M., Tejo B. A., Abedikargiban R., Abdul Rahman M. B. A., Micaêlo N. M. (2014). Modeling stability and flexibility of α-Chymotrypsin in room temperature ionic liquids. *Journal of Biomolecular Structure and Dynamics*.

[21] Kumar A., Venkatesu P. (2014). Does the stability of proteins in ionic liquids obey the Hofmeister series?. *International Journal of Biological Macromolecules*.

[22] Khoshtariya D. E., Dolidze T. D., Tretyakova T., Waldeck D. H., van Eldik R. (2013). Electron transfer with azurin at Au/SAM junctions in contact with a protic ionic melt: impact of glassy dynamics. *Physical Chemistry and Chemical Physics*.

[23] Khoshtariya D. E., Shushanian M., Sujashvili R., Makharadze M., Tabuashvili E., Getashvili G. (2003). Enzymatic activity of α-chymotrypsin in the urea-induced moltenglobule-like state: a combined kinetic/ thermodynamic study. *Journal of Biological Physics and Chemistry*.

[24] Shushanyan M., Sujashvili R., Tabuashvili E., Makharadze M., Getashvili G., Khoshtariya D. E. (2006). Kinetic and thermodynamic manifestations of thermally induced molten-globule-like state of α-chymotrypsin. *Journal of Biological Physics and Chemistry*.

[25] Khoshtariya D. E., Dolidze T. D., Seifert S., Sarauli D., Lee G., van Eldik R. (2006). Kinetic, thermodynamic, and mechanistic patterns for free (unbound) cytochrome c at Au/SAM junctions: impact of electronic coupling, hydrostatic pressure, and stabilizing/denaturing additives. *Chemistry*.

[26] Kumar A., Attri P., Venkatesu P. (2012). Effect of polyols on the native structure of *α*-chymotrypsin: a comparable study. *Thermochimica Acta*.

[27] Privalov P. L., Khechinashvilli N. N. (1974). A thermodynamic approach to the problem of stabilization of globular protein structure: a calorimetric study. *Journal of Molecular Biology*.

[28] Privalov P. L., Potekhin S. A. (1986). Scanning microcalorimetry in studying temperature-induced changes in proteins. *Methods in Enzymology*.

[29] Freire E., van Osdol W. W., Mayorga O. L., Sanchez-Ruiz J. M. (1990). Calorimetrically determined dynamics of complex unfolding transitions in proteins. *Annual Review of Biophysics and Biophysical Chemistry*.

[30] Tretyakova T., Shushanyan M., Partskhaladze T., Makharadze M., van Eldik R., Khoshtariya D. E. (2013). Simplicity within the complexity: bilateral impact of DMSO on the functional and unfolding patterns of *α*-chymotrypsin. *Biophysical Chemistry*.

[31] Makhatadze G. I., Privalov P. L. (1995). Energetics of protein structure. *Advances in Protein Chemistry*.

[32] Makhatadze G. I., Privalov P. L. (1996). On the entropy of protein folding. *Protein Science*.

[33] Privalov P. L. (2007). Thermodynamic problems in structural molecular biology. *Pure and Applied Chemistry*.

[34] Gsponer J., Hopearuoho H., Whittaker S. B. (2006). Determination of an ensemble of structures representing the intermediate state of the bacterial immunity protein Im7. *Proceedings of the National Academy of Sciences of the United States of America*.

[35] la Rosa C., Milardi D., Grasso D., Guzzi R., Sportelli L. (1995). Thermodynamics of the thermal unfolding of azurin. *Journal of Physical Chemistry*.

